# The genome sequence of the Round-winged Muslin, *Thumatha senex *(Hübner, 1804)

**DOI:** 10.12688/wellcomeopenres.19648.1

**Published:** 2023-07-11

**Authors:** Douglas Boyes, Ian Sims, Peter W.H. Holland, University of Oxford and Wytham Woods Genome Acquisition Lab, Natural History Museum Genome Acquisition Lab, Darwin Tree of Life Barcoding collective, Wellcome Sanger Institute Tree of Life programme, Wellcome Sanger Institute Scientific Operations: DNA Pipelines collective, Tree of Life Core Informatics collective, Darwin Tree of Life Consortium

**Affiliations:** 1 UK Centre for Ecology & Hydrology, Wallingford, England, UK; 2 Syngenta UK, Fulbourn, England, UK; 3 University of Oxford, Oxford, England, UK

**Keywords:** Thumatha senex, Round-winged Muslin, genome sequence, chromosomal, Lepidoptera

## Abstract

We present a genome assembly from an individual female *Thumatha senex *(the Round-winged Muslin; Arthropoda; Insecta; Lepidoptera; Erebidae). The genome sequence is 810.3 megabases in span. Most of the assembly is scaffolded into 30 chromosomal pseudomolecules, including the W and Z sex chromosomes. The mitochondrial genome has also been assembled and is 15.5 kilobases in length.

## Species taxonomy

Eukaryota; Metazoa; Eumetazoa; Bilateria; Protostomia; Ecdysozoa; Panarthropoda; Arthropoda; Mandibulata; Pancrustacea; Hexapoda; Insecta; Dicondylia; Pterygota; Neoptera; Endopterygota; Amphiesmenoptera; Lepidoptera; Glossata; Neolepidoptera; Heteroneura; Ditrysia; Obtectomera; Noctuoidea; Erebidae; Arctiinae; Lithosiini; *Thumatha*; *Thumatha senex* (Hübner, 1804) (NCBI:txid997290).

## Background

The Round-winged Muslin, *Thumatha senex*, is a small moth in the subfamily Arctiinae, family Erebidae, closely related to the Footman and Tiger moths. The genus *Thumatha* includes approximately 20 species, most restricted to sub-Saharan Africa; *T. senex* is the only representative found in Europe ( Volynkin, 2021). While many members of the Arctiinae have brightly coloured wings, advertising unpalatability, *T. senex* has grey-brown wings with a thin covering of scales giving the moth a papery, translucent and delicate appearance.

In Britain, the moth is widely distributed in marshy areas, fenland and damp woodland in the south-east of England especially East Anglia, but it is less common in central and northern England, Wales and Northern Ireland; it is scarce in Scotland ( NBN Atlas Partnership, 2021). In Ireland, the moth has been recorded from central and eastern regions ( MothsIreland, 2022). In mainland Europe, the species is also associated with wetland habitats, with many records from the Netherlands, Scandinavia and France; there are scattered records further east across Eurasia including from Russia ( GBIF Secretariat, 2022). The larvae are usually described as moss and lichen feeders although a study in marshy ground in southern Germany found the larvae more commonly in the detritus layer feeding on decaying vegetation from sedge *Carex* sp. ( Wagner, 2023). The adults, which fly in summer, have been described as swarming in large numbers at dusk on warm, still nights in suitable habitats ( South, 1961; Wagner, 2023).

The genome sequence of *Thumatha senex* was determined as part of the Darwin Tree of Life project. The assembled genome sequence will facilitate research into adaptations to wetland habitats and contribute to the growing set of resources for studying insect ecology and evolution.

## Genome sequence report

**Figure 1.**
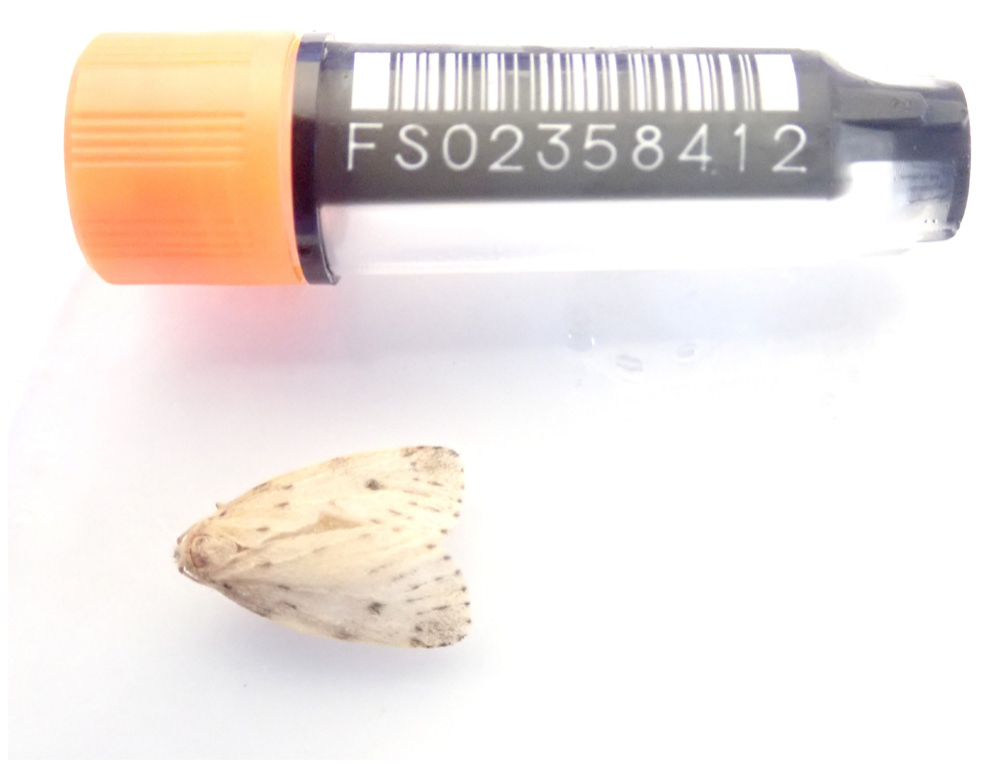
Photograph of the *Thumatha senex* (ilThuSene1) specimen used for genome sequencing.

The genome was sequenced from one female *Thumatha senex* ( Figure 1) collected from Wytham Woods, Oxfordshire, UK (51.77, –1.34). A total of 48-fold coverage in Pacific Biosciences single-molecule HiFi long reads was generated. Primary assembly contigs were scaffolded with chromosome conformation Hi-C data. Manual assembly curation corrected 11 missing joins or mis-joins and removed two haplotypic duplications.

**Figure 2.**
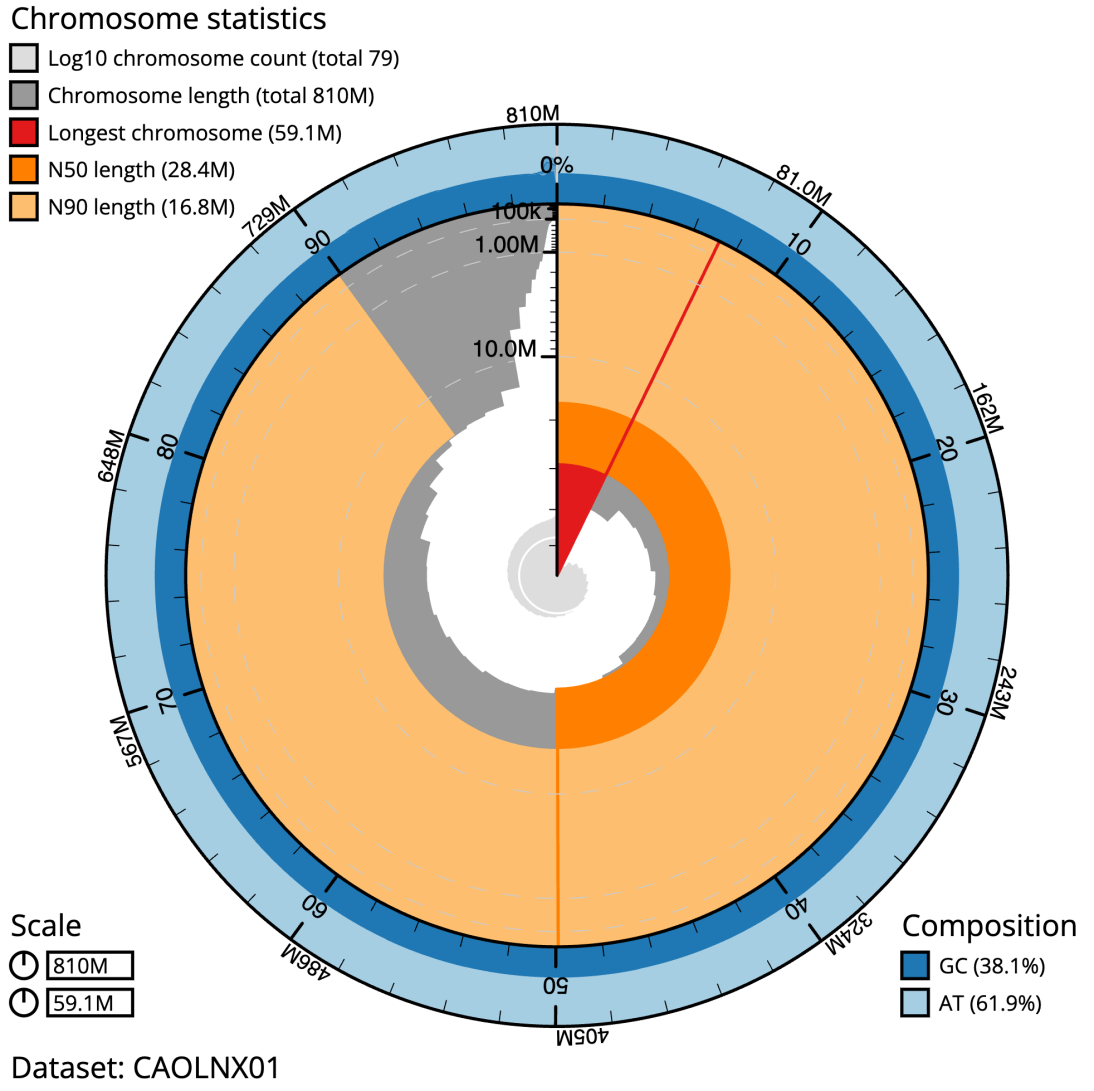
Genome assembly of *Thumatha senex*, ilThuSene1.1: metrics. The BlobToolKit Snailplot shows N50 metrics and BUSCO gene completeness. The main plot is divided into 1,000 size-ordered bins around the circumference with each bin representing 0.1% of the 810,282,324 bp assembly. The distribution of scaffold lengths is shown in dark grey with the plot radius scaled to the longest scaffold present in the assembly (59,070,461 bp, shown in red). Orange and pale-orange arcs show the N50 and N90 scaffold lengths (28,419,001 and 16,758,000 bp), respectively. The pale grey spiral shows the cumulative scaffold count on a log scale with white scale lines showing successive orders of magnitude. The blue and pale-blue area around the outside of the plot shows the distribution of GC, AT and N percentages in the same bins as the inner plot. A summary of complete, fragmented, duplicated and missing BUSCO genes in the lepidoptera_odb10 set is shown in the top right. An interactive version of this figure is available at https://blobtoolkit.genomehubs.org/view/Thumatha senex/dataset/CAOLNX01/snail.

The final assembly has a total length of 810.3 Mb in 78 sequence scaffolds with a scaffold N50 of 28.4 Mb ( Table 1). Most (99.48%) of the assembly sequence was assigned to 30 chromosomal-level scaffolds, representing 28 autosomes and the W and Z sex chromosomes. Chromosome-scale scaffolds confirmed by the Hi-C data are named in order of size ( Figure 2– Figure 5; Table 2). The order and orientation of W chromosome contigs is unknown as the Hi-C data used for scaffolding was derived from a male sample (PacBio HiFi data used for *de novo* assembly is from a female sample). While not fully phased, the assembly deposited is of one haplotype. Contigs corresponding to the second haplotype have also been deposited. The mitochondrial genome was also assembled and can be found as a contig within the multifasta file of the genome submission.

**Table 1.** Genome data for *Thumatha senex*, ilThuSene1.1.

Project accession data		
Assembly identifier	ilThuSene1.1	
Species	*Thumatha senex*	
Specimen	ilThuSene1	
NCBI taxonomy ID	997290	
BioProject	PRJEB59089	
BioSample ID	SAMEA7701482	
Isolate information	ilThuSene1, female: whole organism (DNA sequencing) ilThuSene2, male: head and thorax (Hi-C scaffolding)	
Assembly metrics *		*Benchmark*
Consensus quality (QV)	66.2	*≥ 50*
*k*-mer completeness	100%	*≥ 95%*
BUSCO **	C:98.5%[S:97.8%,D:0.7%], F:0.4%,M:1.1%,n:5,286	*C ≥ 95%*
Percentage of assembly mapped to chromosomes	99.48%	*≥ 95%*
Sex chromosomes	W and Z chromosomes	*localised* * homologous* * pairs*
Organelles	Mitochondrial genome assembled	*complete* * single alleles*
Raw data accessions		
PacificBiosciences SEQUEL II	ERR10798436, ERR10798437, ERR10802390		
Hi-C Illumina	ERR10802464		
Genome assembly		
Assembly accession	GCA_948477245.1	
*Accession of alternate* * haplotype*	GCA_948576625.1	
Span (Mb)	810.3	
Number of contigs	89	
Contig N50 length (Mb)	27.3	
Number of scaffolds	78	
Scaffold N50 length (Mb)	28.4	
Longest scaffold (Mb)	59.1	

* Assembly metric benchmarks are adapted from column VGP-2020 of “Table 1: Proposed standards and metrics for defining genome assembly quality” from ( Rhie *et al.*, 2021). ** BUSCO scores based on the lepidoptera_odb10 BUSCO set using v5.3.2. C = complete [S = single copy, D = duplicated], F = fragmented, M = missing, n = number of orthologues in comparison. A full set of BUSCO scores is available at https://blobtoolkit.genomehubs.org/view/Thumatha senex/dataset/CAOLNX01/busco.

**Figure 3.**
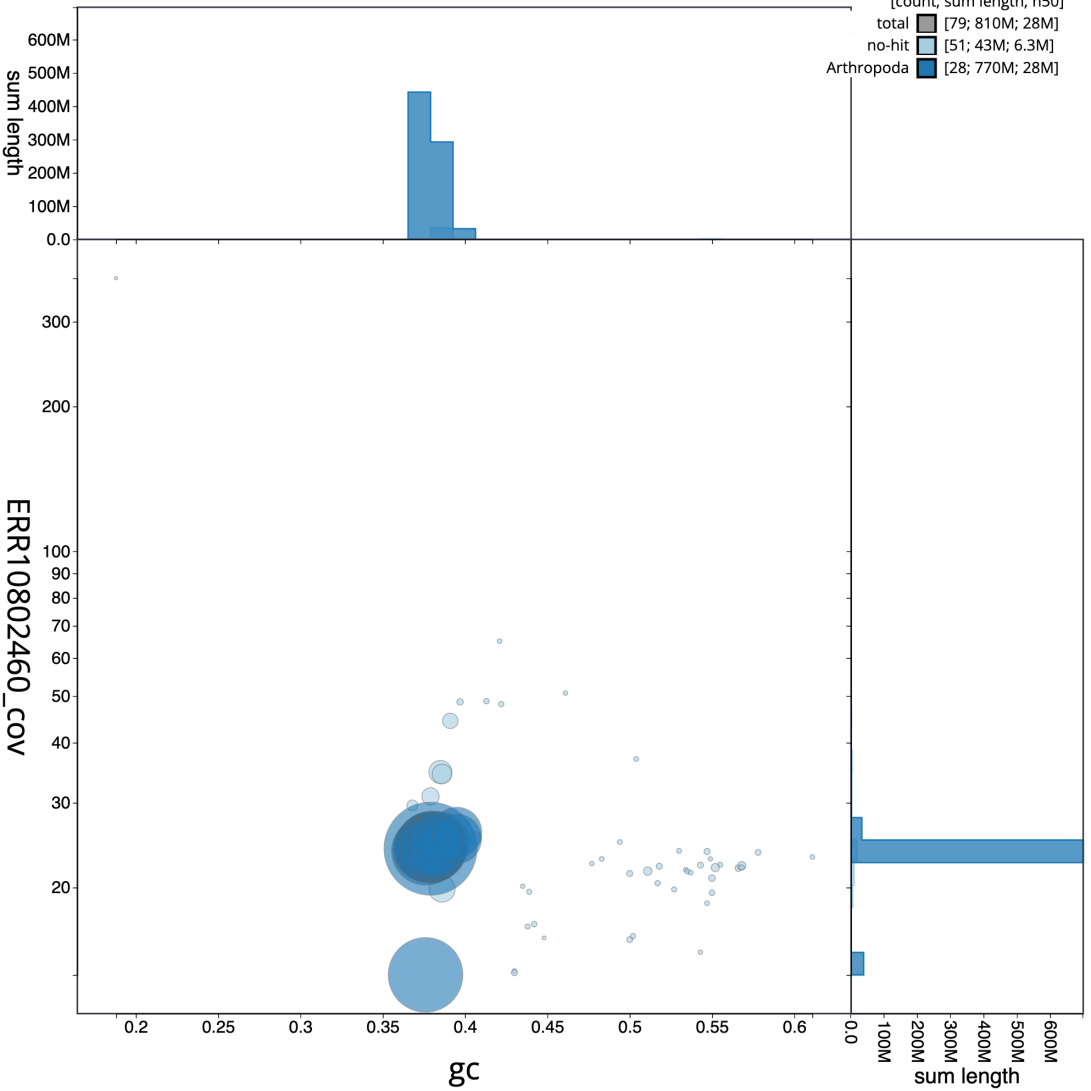
Genome assembly of *Thumatha senex*, ilThuSene1.1: BlobToolKit GC-coverage plot. Scaffolds are coloured by phylum. Circles are sized in proportion to scaffold length. Histograms show the distribution of scaffold length sum along each axis. An interactive version of this figure is available at https://blobtoolkit.genomehubs.org/view/Thumatha senex/dataset/CAOLNX01/blob.

**Figure 4.**
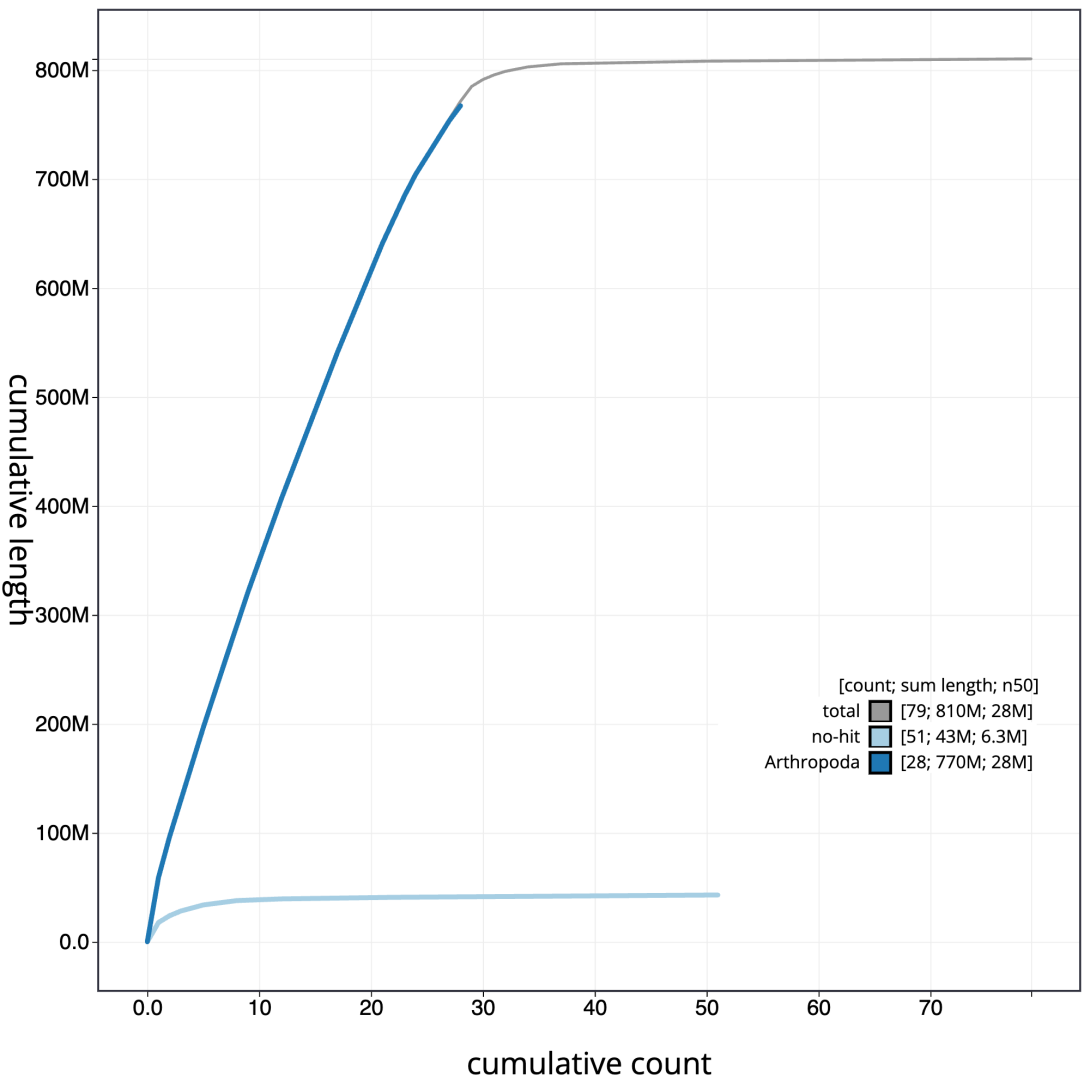
Genome assembly of *Thumatha senex*, ilThuSene1.1: BlobToolKit cumulative sequence plot. The grey line shows cumulative length for all scaffolds. Coloured lines show cumulative lengths of scaffolds assigned to each phylum using the buscogenes taxrule. An interactive version of this figure is available at https://blobtoolkit.genomehubs.org/view/Thumatha senex/dataset/CAOLNX01/cumulative.

**Figure 5.**
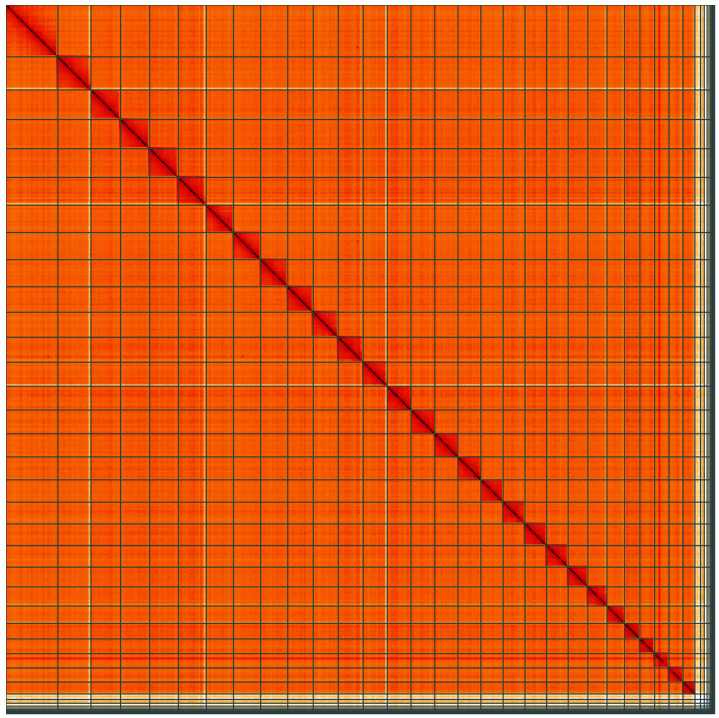
Genome assembly of *Thumatha senex*, ilThuSene1.1: Hi-C contact map of the ilThuSene1.1 assembly, visualised using HiGlass. Chromosomes are shown in order of size from left to right and top to bottom. An interactive version of this figure may be viewed at https://genome-note-higlass.tol.sanger.ac.uk/l/?d=ZmhhH1o7TiCmqSVOtyNZcg.

**Table 2.** Chromosomal pseudomolecules in the genome assembly of *Thumatha senex*, ilThuSene1.

INSDC accession	Chromosome	Length (Mb)	GC%
OX419724.1	1	59.07	38.0
OX419726.1	2	33.73	38.0
OX419727.1	3	33.22	38.0
OX419728.1	4	32.76	38.0
OX419729.1	5	31.67	38.0
OX419730.1	6	31.05	37.5
OX419731.1	7	30.98	38.0
OX419732.1	8	30.86	38.0
OX419733.1	9	29.09	37.5
OX419734.1	10	28.47	37.5
OX419735.1	11	28.42	38.0
OX419736.1	12	27.33	38.0
OX419737.1	13	27.16	38.0
OX419738.1	14	27.03	37.5
OX419739.1	15	26.36	38.0
OX419740.1	16	26.14	38.0
OX419741.1	17	25.43	38.0
OX419742.1	18	24.87	38.5
OX419743.1	19	24.71	38.0
OX419744.1	20	24.54	38.0
OX419745.1	21	22.42	38.0
OX419746.1	22	21.76	38.5
OX419748.1	23	19.76	38.0
OX419749.1	24	17.75	38.5
OX419750.1	25	16.76	38.5
OX419751.1	26	16.4	39.5
OX419752.1	27	15.94	39.5
OX419753.1	28	13.67	38.5
OX419747.1	W	6.26	38.5
OX419725.1	Z	37.69	37.5
OX419754.1	MT	0.02	19.0

The estimated Quality Value (QV) of the final assembly is 66.2 with *k*-mer completeness of 100%, and the assembly has a BUSCO v5.3.2 completeness of 98.5% (single = 97.8%, duplicated = 0.7%), using the lepidoptera_odb10 reference set ( *n* = 5,286).

Metadata for specimens, spectral estimates, sequencing runs, contaminants and pre-curation assembly statistics can be found at https://links.tol.sanger.ac.uk/species/997290.

## Methods

### Sample acquisition and nucleic acid extraction

The specimen used for genome sequencing was a female *Thumatha senex* (specimen number Ox000618, individual ilThuSene1) was collected from Wytham Woods, Oxfordshire (biological vice-county Berkshire), UK (latitude 51.77, longitude –1.34) on 2020-07-05, using a light trap. Douglas Boyes (University of Oxford) collected and identified the specimen. The specimen was snap-frozen on dry ice.

A male *T. senex* specimen (specimen number NHMUK013805987, ilThuSene2) was collected from Hartslock Nature Reserve (latitude 51.51, longitude –1.11) on 2021-07-29. The specimen was collected and identified by Ian Sims (British Entomological and Natural History Society). This specimen was used for Hi-C scaffolding. 

DNA was extracted at the Tree of Life laboratory, Wellcome Sanger Institute (WSI). The ilThuSene1 sample was weighed and dissected on dry ice with tissue set aside for Hi-C sequencing. Whole organism tissue was disrupted using a Nippi Powermasher fitted with a BioMasher pestle. High molecular weight (HMW) DNA was extracted using the Qiagen MagAttract HMW DNA extraction kit. HMW DNA was sheared into an average fragment size of 12–20 kb in a Megaruptor 3 system with speed setting 30. Sheared DNA was purified by solid-phase reversible immobilisation using AMPure PB beads with a 1.8X ratio of beads to sample to remove the shorter fragments and concentrate the DNA sample. The concentration of the sheared and purified DNA was assessed using a Nanodrop spectrophotometer and Qubit Fluorometer and Qubit dsDNA High Sensitivity Assay kit. Fragment size distribution was evaluated by running the sample on the FemtoPulse system.

### Sequencing

Pacific Biosciences HiFi circular consensus DNA sequencing libraries were constructed according to the manufacturers’ instructions. DNA sequencing was performed by the Scientific Operations core at the WSI on the Pacific Biosciences SEQUEL II (HiFi) instrument. Hi-C data were also generated from head and thorax tissue of ilThuSene2 using the Arima2 kit and sequenced on the Illumina NovaSeq 6000 instrument.

### Genome assembly, curation and evaluation

Assembly was carried out with Hifiasm ( Cheng *et al.*, 2021) and haplotypic duplication was identified and removed with purge_dups ( Guan *et al.*, 2020). The assembly was scaffolded with Hi-C data ( Rao *et al.*, 2014) using YaHS ( Zhou *et al.*, 2023). The assembly was checked for contamination and corrected as described previously ( Howe *et al.*, 2021). Manual curation was performed using HiGlass ( Kerpedjiev *et al.*, 2018) and Pretext ( Harry, 2022). The mitochondrial genome was assembled using MitoHiFi ( Uliano-Silva *et al.*, 2022), which runs MitoFinder ( Allio *et al.*, 2020) or MITOS ( Bernt *et al.*, 2013) and uses these annotations to select the final mitochondrial contig and to ensure the general quality of the sequence.

A Hi-C map for the final assembly was produced using bwa-mem2 ( Vasimuddin *et al.*, 2019) in the Cooler file format ( Abdennur & Mirny, 2020). To assess the assembly metrics, the *k*-mer completeness and QV consensus quality values were calculated in Merqury ( Rhie *et al.*, 2020). This work was done using Nextflow ( Di Tommaso *et al.*, 2017) DSL2 pipelines “sanger-tol/readmapping” ( Surana *et al.*, 2023a) and “sanger-tol/genomenote” ( Surana *et al.*, 2023b). The genome was analysed within the BlobToolKit environment ( Challis *et al.*, 2020) and BUSCO scores ( Manni *et al.*, 2021; Simão *et al.*, 2015) were calculated.

Table 3 contains a list of relevant software tool versions and sources.

**Table 3.** Software tools: versions and sources.

Software tool	Version	Source
BlobToolKit	4.1.5	https://github.com/blobtoolkit/blobtoolkit
BUSCO	5.3.2	https://gitlab.com/ezlab/busco
Hifiasm	0.16.1-r375	https://github.com/chhylp123/hifiasm
HiGlass	1.11.6	https://github.com/higlass/higlass
Merqury	MerquryFK	https://github.com/thegenemyers/MERQURY.FK
MitoHiFi	2	https://github.com/marcelauliano/MitoHiFi
PretextView	0.2	https://github.com/wtsi-hpag/PretextView
purge_dups	1.2.3	https://github.com/dfguan/purge_dups
sanger-tol/genomenote	v1.0	https://github.com/sanger-tol/genomenote
sanger-tol/readmapping	1.1.0	https://github.com/sanger-tol/readmapping/tree/1.1.0
YaHS	1.2a	https://github.com/c-zhou/yahs

### Wellcome Sanger Institute - Legal and Governance

The materials that have contributed to this genome note have been supplied by a Darwin Tree of Life Partner. The submission of materials by a Darwin Tree of Life Partner is subject to the **‘Darwin Tree of Life Project Sampling Code of Practice’**, which can be found in full on the Darwin Tree of Life website here. By agreeing with and signing up to the Sampling Code of Practice, the Darwin Tree of Life Partner agrees they will meet the legal and ethical requirements and standards set out within this document in respect of all samples acquired for, and supplied to, the Darwin Tree of Life Project.

Further, the Wellcome Sanger Institute employs a process whereby due diligence is carried out proportionate to the nature of the materials themselves, and the circumstances under which they have been/are to be collected and provided for use. The purpose of this is to address and mitigate any potential legal and/or ethical implications of receipt and use of the materials as part of the research project, and to ensure that in doing so we align with best practice wherever possible. The overarching areas of consideration are:

- Ethical review of provenance and sourcing of the material
- Legality of collection, transfer and use (national and international)

Each transfer of samples is further undertaken according to a Research Collaboration Agreement or Material Transfer Agreement entered into by the Darwin Tree of Life Partner, Genome Research Limited (operating as the Wellcome Sanger Institute), and in some circumstances other Darwin Tree of Life collaborators.

## Data Availability

European Nucleotide Archive: *Thumatha senex* (round-winged muslin). Accession number PRJEB59089; https://identifiers.org/ena.embl/PRJEB59089. ( Wellcome Sanger Institute, 2023) The genome sequence is released openly for reuse. The *Thumatha senex* genome sequencing initiative is part of the Darwin Tree of Life (DToL) project. All raw sequence data and the assembly have been deposited in INSDC databases. The genome will be annotated using available RNA-Seq data and presented through the Ensembl pipeline at the European Bioinformatics Institute. Raw data and assembly accession identifiers are reported in Table 1.

## Author information

Members of the University of Oxford and Wytham Woods Genome Acquisition Lab are listed here: https://doi.org/10.5281/zenodo.4789928.

Members of the Natural History Museum Genome Acquisition Lab are listed here: https://doi.org/10.5281/zenodo.4790042.

Members of the Darwin Tree of Life Barcoding collective are listed here: https://doi.org/10.5281/zenodo.4893703.

Members of the Wellcome Sanger Institute Tree of Life programme are listed here: https://doi.org/10.5281/zenodo.4783585.

Members of Wellcome Sanger Institute Scientific Operations: DNA Pipelines collective are listed here: https://doi.org/10.5281/zenodo.4790455.

Members of the Tree of Life Core Informatics collective are listed here: https://doi.org/10.5281/zenodo.5013541.

Members of the Darwin Tree of Life Consortium are listed here: https://doi.org/10.5281/zenodo.4783558.
