# Supplementary material for: The genome sequence of the Round-winged Muslin, Thumatha senex (Hübner, 1804)
Source: Wellcome Open Res. 2023 Jul 11;8:298. [Version 1] doi: 10.12688/wellcomeopenres.19648.1 (PMC10472059; doi:10.12688/wellcomeopenres.19648.1)
Supplement: Peer review history

# Peer review: Reviewer response for version 1

**DOI:** 10.21956/wellcomeopenres.21765.r64906

**Published:** 2023-08-31

**Authors:** Kuppusamy Sivasankaran

**Affiliations:** 1 Division of Taxonomy and Biodiversity, Entomology Research Institute, Loyola College, Chennai, Tamil Nadu, India

I appreciate the authors for compiling *Thumatha senex's* whole genome sequence. They employed the appropriate software, genome assembly techniques for the whole genome sequencing. 

**Minor changes in the manuscript**

**In the Methods**

- In the first paragraph, the first sentence can be modified as “A female *Thumatha senex *(specimen number Ox000618) moth specimen was collected from Wytham Woods, Oxforshire (biological vice-country Berkshire), UK (latitude 51.77 longitude -1.34) on 2020-07-05, using a light trap. Douglas Boyes (University of Oxford) collected and identified the moth species. The specimen was snap-frozen on dry ice until the nucleic acid extraction."
- In the third paragraph the first sentence starts as “The DNA was extracted..." but the second sentence starts as “The whole organism...”?
- **Query**: Why have the authors used male and female specimens for the DNA extraction?

Overall, the manuscript is written well and it can be Approved for Indexing.

Are sufficient details of methods and materials provided to allow replication by others?

Yes

Is the rationale for creating the dataset(s) clearly described?

Yes

Are the datasets clearly presented in a useable and accessible format?

Yes

Are the protocols appropriate and is the work technically sound?

Yes

Reviewer Expertise:

Taxonomy and phylogenetic implications of superfamily Noctuoidea moths using mitochondrial genome sequence.

I confirm that I have read this submission and believe that I have an appropriate level of expertise to confirm that it is of an acceptable scientific standard.

# Peer review: Reviewer response for version 1

**DOI:** 10.21956/wellcomeopenres.21765.r64912

**Published:** 2023-08-17

**Authors:** Nicolas Dowdy, Kathryn Sullivan, Makani Fisher

**Affiliations:** 1 Zoology, Milwaukee Public Museum, Milwaukee, Wisconsin, USA; 2 Department of Biology, Wake Forest University, Winston-Salem, North Carolina, USA; 3 Biological Sciences, Marquette University, Milwaukee, Wisconsin, USA; 4 Entomology, Purdue University, West Lafayette, Indiana, USA

This is a fantastic addition to the genomic resources of the Lepidoptera of Britain and Ireland. I have just a few notes, including some figure errors that require adjustment:

- Remove "closely related to the Footman and Tiger moths". It is more common for 'tiger moths' to refer to members of Arctiinae (and so Thumatha is a member of the group) and in some cases "Footman" can refer to Lithosiini, to which Thumatha is also a member.
- "approximately 20 species" is more accurately "13 species currently".
- Is/was the specimen deposited? Is there a voucher remaining?
- Some justification as to why a male was used for HiFi instead of a female would be welcomed.
- Illumina NovaSeq 6000 read archive information is missing from the "Raw Data Accessions" table, but used to generate Figure 3.
- Based on the GenBank WGS Project page ( https://www.ncbi.nlm.nih.gov/nuccore/CAOLNX000000000.1) it appears there are 8 associated sequence read archives, but only 4 are included in the "Raw Data Accessions" table. Is there a reason for that?
- Fig. 2 missing the BUSCO graph described in figure caption.
- Fig. 3 partly cuts off.

Are sufficient details of methods and materials provided to allow replication by others?

Yes

Is the rationale for creating the dataset(s) clearly described?

Yes

Are the datasets clearly presented in a useable and accessible format?

Yes

Are the protocols appropriate and is the work technically sound?

Yes

Reviewer Expertise:

Phylogenomics, evolution, and behavioral ecology of Noctuiodea, especially Erebidae and Arctiinae.

We confirm that we have read this submission and believe that we have an appropriate level of expertise to confirm that it is of an acceptable scientific standard, however we have significant reservations, as outlined above.

# Peer review: Reviewer response for version 1

**DOI:** 10.21956/wellcomeopenres.21765.r63692

**Published:** 2023-08-01

**Authors:** Maria Cristina Arias

**Affiliations:** 1 Instituto de Biociências, University of Sao Paulo, São Paulo, State of São Paulo, Brazil

The paper reports the genome sequence of *Thumatha senex,* a Lepidoptera *. *The genome now available will enable to prospect it for putative genes or genome regions related to adaptations to wetlands. The paper is presented as DATA NOTE and follows the standard writing for this category. All the details concerning methods, software, dataset and their respective links are available. Thus I fully recommend this paper for indexing.

Are sufficient details of methods and materials provided to allow replication by others?

Yes

Is the rationale for creating the dataset(s) clearly described?

Yes

Are the datasets clearly presented in a useable and accessible format?

Yes

Are the protocols appropriate and is the work technically sound?

Yes

Reviewer Expertise:

Genetics and evoluiton of bees

I confirm that I have read this submission and believe that I have an appropriate level of expertise to confirm that it is of an acceptable scientific standard.
